# Flexor tenotomies for the treatment of bilateral wrist contracture after traumatic brain injury: A case report

**DOI:** 10.1002/ccr3.7869

**Published:** 2023-09-22

**Authors:** Elizabeth Brown, Tyler Kastner, Justin Harder, Cameron Cox, Brendan MacKay

**Affiliations:** ^1^ Texas Tech University Health Sciences Center Lubbock Texas USA; ^2^ Department of Orthopaedic Hand Surgery Texas Tech University Health Sciences Center Lubbock Texas USA

**Keywords:** contracture, orthopedics, surgery, tenotomy

## Abstract

Traumatic brain injuries have the potential to cause the development of long‐term complications. We aim to show that the use of flexor tenotomies in the treatment of flexion contractures following traumatic brain injury is a viable solution.

## 1 INTRODUCTION

Traumatic brain injury (TBI) is a common result of falls and collisions, such as motor vehicle accidents, in millions of patients annually.^1^ Injuries of this type are associated with an abundance of long‐term complications such as Parkinson's disease, Alzheimer's disease, dementia pugilistica, psychiatric disorders, and posttraumatic epilepsy as well as cognitive and behavioral difficulties.^2^ Primary treatment of TBI varies based on the severity of the injury. Treatment modalities may range from cognitive therapy to surgical procedures to achieve restoration of blood flow and removal of mass lesions.^1^ However, long‐term treatment involves addressing specific deficits, such as musculoskeletal and neurological damage, directly through therapeutic approaches.^3^ If not addressed, TBI may leave patients with debilitating long‐term disabilities as a result of such muscle and nerve impairment.^4^

Musculoskeletal deficiency as a result of TBI may stem from upper motor neuron damage that could be the result of a lesion.^5^, ^6^, ^7^, ^8^ While surgical intervention for treatment of such lesions includes few and specific diagnoses such as intracranial aneurysms, hematomas, and malignancies, the sequelae of damage may be addressed.^9^ Complications of this kind may result in musculoskeletal overactivity or deficits which may lead to problems with utilization of the affected limb.^8^ Common muscular overactivity associated with TBI is flexion contracture of the limbs secondary to adaptive shortening of soft tissues spanning joints.^10^ This can lead to serious functional deficits such as a lack of full passive range of motion associated with decreased motor recovery and difficulty performing regular functions of daily life.^11^ Mainstay treatment for flexion contractures is nonoperative management with passive stretching through serial casting, splints, and physical therapy. While these nonoperative measures have been successful in pediatric patients with congenital deformities and in some cases after soft tissue injury, they have not been well examined in other populations.^12^, ^13^, ^14^ Surgical treatment for contracture following TBI, although potentially beneficial, has not been addressed thoroughly in the literature.^15^

A few studies have reported on surgical treatments for flexor tendon contracture, especially in the knee. Schnitzler, et al. performed a retrospective study treating knee flexion contracture with percutaneous needle tenotomy for tendon lengthening.^16^ Overall, the treatment was well tolerated with significantly increased range of motion. Another study evaluated flexion contractures after total knee arthroplasty and found success with bone resection and subsequent posterior capsule and collateral ligament release.^17^ Additionally, a handful of case reports have also noted success in surgical approach to upper extremity flexion contracture seen after central nervous system injury. Z‐lengthening, fractional lengthening of the tendon, as well as myotomy have been found to improve range of motion and pain in patients with spastic contracture of the upper extremities after brain injury.^15^, ^18^, ^19^, ^20^, ^21^, ^22^, ^23^

Due to few case reports being noted in the literature and unclear consensus toward the treatment protocol for such an injury, we aim to show that the use of flexor tenotomies in the treatment of flexion contractures following TBI is a viable solution. We report an 18‐year‐old male with bilateral upper extremity flexion contractures after a TBI, treated surgically by release and lengthening of the tendons.

## 2 CASE REPORT

An 18‐year‐old African American male sustained bilateral wrist flexion contractures after a closed head injury playing football. While he was not formally diagnosed with a concussion, the force was enough to detach his retina. The patient did not note any additional neurological issues such as sleep disruption or difficulty with balance. He had acute onset limitation of active extension of his wrist that did not limit his day to day school involvement. The patient attended hand therapy to address the limitation in extension of his wrist. As therapy was not effective at treating his deformities and caused discomfort, the patient was referred to our clinic.

Patient presented to clinic 2 years following his injury. At initial examination, the patient was noted to have boutonniere deformities of Fingers 3 and 4 on his left hand. Extension of the bilateral wrists were 10 degrees with discomfort on passive manipulation. Due to concern that these deformities may be of central neurologic origin, an MRI and bilateral upper extremity EMG were performed. The results of these tests were reviewed and displayed mixed nerve latency, distal motor latency, sensory and motor amplitude, and conduction velocity to be within standard values bilaterally. While EMG results ruled out neural irregularity, the muscular involvement and time of presentation following injury indicated neuromuscular origin. Spasticity as a result of upper motor neuron damage was suspected. Due to continuous display of a tight contracture, he was sent to further therapy for continued range of motion and strengthening at the digits and wrist with splinting of his left‐hand Fingers 3 and 4.

A year later, the patient returned to clinic and displayed similar findings as previous exams. As a result of failure of physical therapy and splinting due to pain, the patient chose to pursue operative management (left upper extremity flexor tendon lengthening).

A 15 cm Bruner's incision was made over the anterior aspect of the left forearm, over the radius. The fascia overlying the flexor carpi radialis (FCR), palmaris, brachioradialis, flexor digitorum superficialis (FDS), and flexor digitorum profundus (FDP) was noted to have dense adhesions and fibrous tissue that was released and debrided. An exam under anesthesia found limitation of range of motion that was consistent with the limitations present during previous assessments, ruling out muscular dystonia. It was observed that the tendons were tight and restricting the motion at the wrist, long finger, and ring finger. At this time a 15‐blade was used to lengthen the muscle bellies of FCR, palmaris, brachioradialis, FDS, and FDP. These tendons were chosen to be released as their contraction was noted to have effect on the patient's range of motion upon intraoperative exam. Range of motion was then found to be significantly improved. The incisions were closed and a volar splint was placed with the left long and ring finger proximal interphalangeal joints in extension for treatment of the pseudo‐boutonniere deformity.

Postoperatively, the patient participated in 2 months of physical therapy for his left hand with identical range of motion and strengthening orders as preoperative therapy. These orders were not altered as they are standard following tendon release and provide the patient with the best opportunity for rehabilitation. While postoperative recurrence of the contractures could not be predicted, physical therapy and routine stretching were implemented as preventative measures in the postoperative plan. At 4 months postoperatively, the patient was content with the improved function of his left wrist and hand along with experiencing no pain. His wrist extension was improved to nearly full active range of motion with an extension deficit of about 10 degrees. However, his pseudo‐boutonniere deformity to the left long finger was still present at this time. Due to satisfactory improvement in function and pain, the patient requested flexor tendon lengthening of his right wrist.

On passive manipulation, the right wrist was noted to have full wrist flexion, but wrist extension was 20 degrees with discomfort, similar to the left before surgical intervention. Fractional lengthening of brachioradialis, FCR, palmaris longus, flexor pollicis, FDS, FDP, and flexor carpi ulnaris was performed. Exam under anesthesia was then performed, ensuring the patient could obtain full extension of all digits and the wrist.

Following bilateral tenotomies, the patient had noted improvement in range of motion bilaterally and was able to perform activities of daily life that he could not perform since his injury.

## 3 DISCUSSION

Musculoskeletal symptoms are a common complication of traumatic brain injury. Our patient presented with bilateral upper extremity flexion contractures after his TBI. Treatment options for flexion contracture after TBI remains to be a debated and unclear topic.

Conservative, noninvasive treatment for flexion contracture after TBI should be utilized in most cases as the first‐treatment option. Serial casting with intensive physical therapy, with or without muscle relaxants, are the most common practice. Several case studies have reported improvement in range of motion and mobility following serial casting which involves progressive, fixed extension in a fiberglass cast.^10^, ^24^, ^25^, ^26^, ^27^ However, another study by Moseley, et al. showed only a transient improvement in contracture after serial casting with almost complete reversion back to contracture by the 4‐week follow‐up.^28^ Several studies aimed to improve the therapeutic use of serial casting by adding muscle relaxants such as botulinum toxin or baclofen and intensive physical therapy to the treatment protocol; however, there still does not seem to be a tried and true method for long‐term correction of flexion contracture after TBI.^15^, ^29^ Many flexion contracture cases treated with conservative management were contractures of the lower extremity, mainly the ankle and the knee with few cases highlighting success of conservative management of upper extremity flexion contracture. These options proved unsuccessful in our case preoperatively due to the patient's pain upon attempting therapy and inability to consistently attend as a result. It could be concluded that since the contractures had been present in the patient for over 2 years without consistent therapy, they were likely fixed and therefore would not respond to stretching and splinting.^11^ As a result, further intervention had to be considered.

Some studies have proposed surgical treatment for flexion contractures after TBI and have noted improvement postoperatively. It has been suggested that flexion contracture caused by upper motor neuron damage, which failed nonoperative stretching, may be released by excising the muscle or performing a Z‐lengthening to return the length.^18^ Several studies have demonstrated success through improved active and passive range of motion, decreased pain, and increased functionality after surgical correction of flexion contractures due to upper motor neuron damage.^18^, ^19^, ^20^, ^21^, ^22^, ^23^, ^30^ The majority of these studies involved upper extremity flexion contractures; however, a few lower extremity contractures were treated with surgical intervention. Our patient demonstrated improved functionality and range of motion after his bilateral upper extremity tenotomies, highlighting the success of surgical management for spastic upper extremities following brain injury.

This case demonstrates the potential for success when utilizing this surgical method in the upper extremities. The return of function experienced by the patient in our study was much greater than that of nonoperative treatment due to the unique circumstances with which our patient presented. Although each case varies, we propose that despite the obvious risks associated with surgical treatment, the benefits have the potential to outweigh the risks and help the patient have better outcomes.

## 4 CONCLUSION

Proper treatment of flexion contractures after traumatic brain injury has sparked debate between physicians as to which technique should be utilized. While nonoperative management has proven successful in some cases, it is not always the best option due to the underlying spasticity caused by upper motor neuron damage. Through this study, we found that our patient experienced significant improvement in range of motion and functionality after tenotomy of his bilateral upper extremity flexion contractures. We believe tenotomy is a viable treatment for flexion contracture after TBI in the right patient population.

### LIMITATIONS

Unfortunately, clinical preoperative and postoperative imaging was not able to be obtained as the patient could not be contacted. We acknowledge that imaging is important to the case and would provide clarification to the procedures performed.

## AUTHOR CONTRIBUTIONS

**Elizabeth Brown:** Writing – original draft; writing – review and editing. **Tyler Kastner:** Writing – original draft. **Justin Harder:** Writing – original draft; writing – review and editing. **Cameron Cox:** Writing – review and editing. **Brendan Mackay:** Writing – original draft; writing – review and editing.

## CONFLICT OF INTEREST STATEMENT

The authors have no conflicts of interest to declare relating to the information within this article.

## CONSENT STATEMENT

Written informed consent was obtained from the patient to publish this report in accordance with the journal's patient consent policy.

## Data Availability

The data that support the findings of this study are available on request from the corresponding author. The data are not publicly available due to privacy or ethical restrictions.
